# Variables with time-varying effects and the Cox model: Some statistical concepts illustrated with a prognostic factor study in breast cancer

**DOI:** 10.1186/1471-2288-10-20

**Published:** 2010-03-16

**Authors:** Carine A Bellera, Gaëtan MacGrogan, Marc Debled, Christine Tunon de Lara, Véronique Brouste, Simone Mathoulin-Pélissier

**Affiliations:** 1 Department of Clinical Epidemiology and Clinical Research, Institut Bergonié, Regional Comprehensive Cancer Centre, Bordeaux, France; 2 Department of Pathology, Institut Bergonié, Regional Comprehensive Cancer Centre, Bordeaux, France; 3 Department of Medical Oncology, Institut Bergonié, Regional Comprehensive Cancer Centre, Bordeaux, France; 4 Department of Surgery, Institut Bergonié, Regional Comprehensive Cancer Centre, Bordeaux, France; 5 Unité INSERM 897, Université Victor Segalen Bordeaux 2, Bordeaux, France

## Abstract

**Background:**

The Cox model relies on the proportional hazards (PH) assumption, implying that the factors investigated have a constant impact on the hazard - or risk - over time. We emphasize the importance of this assumption and the misleading conclusions that can be inferred if it is violated; this is particularly essential in the presence of long follow-ups.

**Methods:**

We illustrate our discussion by analyzing prognostic factors of metastases in 979 women treated for breast cancer with surgery. Age, tumour size and grade, lymph node involvement, peritumoral vascular invasion (PVI), status of hormone receptors (HRec), Her2, and Mib1 were considered.

**Results:**

Median follow-up was 14 years; 264 women developed metastases. The conventional Cox model suggested that all factors but HRec, Her2, and Mib1 status were strong prognostic factors of metastases. Additional tests indicated that the PH assumption was not satisfied for some variables of the model. Tumour grade had a significant time-varying effect, but although its effect diminished over time, it remained strong. Interestingly, while the conventional Cox model did not show any significant effect of the HRec status, tests provided strong evidence that this variable had a non-constant effect over time. Negative HRec status increased the risk of metastases early but became protective thereafter. This reversal of effect may explain non-significant hazard ratios provided by previous conventional Cox analyses in studies with long follow-ups.

**Conclusions:**

Investigating time-varying effects should be an integral part of Cox survival analyses. Detecting and accounting for time-varying effects provide insights on some specific time patterns, and on valuable biological information that could be missed otherwise.

## Background

Survival analysis, or time-to-event data analysis, is widely used in oncology since we are often interested in studying a delay, such as the time from cancer diagnosis or treatment initiation to cancer recurrence or death. Thanks to the improvement of cancer treatments, and the induced longer life expectancy, we observe an increasing number of studies with long follow-up periods. Statistical models to analyze such data should thus adequately account for the increasing duration of follow-ups. The Cox proportional hazards (PH) model allows one to describe the survival time as a function of multiple prognostic factors [1]. This model relies on a fundamental assumption, the proportionality of the hazards, implying that the factors investigated have a constant impact on the hazard - or risk - over time. If time-dependent variables are included without appropriate modeling, the PH assumption is violated. As a result, misleading effect estimates can be derived, and significant effect in the early (or late) follow-up period may be missed. Checking the proportionality of the hazards should thus be an integral part of a survival analysis by a Cox model. The assumption, however, is not systematically verified. In a 1995 review of cancer publications using a Cox model, Altman et al. reported that most studies did not report verifying this assumption [2]; similar findings were reported recently by one of the co-authors of the present work [3].

Although the Cox model has been widely used (more than 25 000 citations since the publication of the original paper by Cox [4]), recent publications suggest a growing interest in the quality of its applications. Special papers in statistics have been published in the oncology literature providing general introductions to survival analysis [5-8]; topics covered included summarizing survival data, testing for a difference between groups, presenting existing statistical models, or assessing the adequacy of a survival model. Others works focused on providing definition of specific survival endpoints [9], or on the quality of reporting of survival events [3].

Assessing whether the assumption of proportional hazards is a central theme in survival analysis, and as such is discussed in several statistical textbooks [10-14] as well as in the general statistical literature [15-18]. To our knowledge however, this topic has been discussed in few medical journals. Importantly, this strong assumption does not seem to be systematically assessed. For illustration, a recent review of clinical trials with primary analyses based on survival end points showed that only one of the 64 papers that used a Cox model mentioned verifying the PH assumption [3].

Our objective is to inform clinicians, as well as those who read and write manuscripts in medical journals, about the importance of the underlying PH assumption, the misleading conclusions that can be inferred if it is violated, as well as the additional information provided by verifying it. After a theoretical introduction, we describe techniques to assess if this assumption is violated, and model strategies to account for, and describe time-dependency. We illustrate our discussion with a study on prognostic factors in breast cancer.

## Methods and results

### Survival analysis

In many studies, the primary variable of interest is a delay, such as the time from cancer diagnosis to a particular event of interest. This event may be death, and for this reason the analysis of such data is often referred to as survival analysis. The event of interest may not have occurred at the time of the statistical analysis, and similarly, a subject may be lost to follow-up before the event is observed. In such case, data are said to be censored at the time of the analysis or at the time the patient was lost to follow-up. Censored data still bring some information since although we do not know the exact date of the event, we know that it occurred later than the censoring time.

Both the Kaplan-Meier method and the Cox proportional hazards (PH) model allow one to analyze censored data [1,19], and to estimate the survival probability, S(t), that is the probability that a subject survives beyond some time t. Statistically, this probability is provided by the survival function S(t) = P (T > t), where T is the survival time. The Kaplan Meier method estimates the survival probability non-parametrically, that is, assuming no specific underlying function [19]. Several tests are available to compare the survival distributions across groups, including the log-rank and the Mann-Whitney-Wilcoxon tests [20,21]. The Cox PH model accounts for multiple risk factors simultaneously. It does not posit any distribution, or shape for the survival function, however, the instantaneous incidence rate of the event is modeled as a function of time and risk factors.

The instantaneous hazard rate at time t, also called instantaneous incidence, death, or failure rate, or risk, is the instantaneous probability of experiencing an event at time *t*, given that the event has not occurred yet. It is a rate of event per unit of time, and is allowed to vary over time. Just as the risk of events per unit time, one can make an analogy by considering the speed given by a car speedometer, which represents the distance travelled per unit of time. Suppose, that the event of interest is death, and we are interested in its association with n covariates, X_1_, X_2_, ..., X_n_, then the hazard is given by:(1)

The baseline hazard rate h_0_(t) is an unspecified non-negative function of time. It is the time-dependent part of the hazard and corresponds to the hazard rate when all covariate values are equal to zero. β_1_, β_2_, ..., β_n _are the coefficients of the regression function β_1_x_1 _+ β_2_x_2 _+... β_n_x_n_. Suppose that we are interested in a single covariate then the hazard is:(2)

The hazards for two subjects with covariate values x_1 _and x_2 _are thus given respectively by h_x1_(t) = h_0_(t) exp(βx_1_) and h_x2_(t) = h_0_(t) exp(βx_2_), and the hazard ratio is expressed as:(3)

Taking x_2 _= x_1 _+ 1, the hazard ratio reduces to HR = exp(β) and corresponds to the effect of one unit increase in the explanatory variable X on the risk of event. Since β = log(HR), β is referred as the log hazard ratio. Although the hazard rate h_x_(t) is allowed to vary over time, the hazard ratio HR is constant; this is the *assumption of proportional hazards*. If the HR is greater than 1 (β > 0), the event risk is increased for subjects with covariate value x_2 _compared to subjects with covariate value x_1_, while a HR lower than 1 (β < 0) indicates a decreased risk. When the HR is not constant over time, the variable is said to have a time-varying effect; for example, the effect of a treatment can be strong immediately after treatment but fades with time. This should not be confused with a time-varying covariate, which is a variable whose value is not fixed over time, such as smoking status. Indeed, a person can be a non-smoker, then a smoker, then a non-smoker. Note however, that a variable may be both time-varying and have an effect that changes over time.

In a Cox PH model, the HR is estimated by considering each time *t *at which an event occurs. When estimating the overall HR over the complete follow-up period, the same weights are given to the very early HR which affect almost all individuals and to very late HR affecting only the very few individuals still at risk. The HR is thus *averaged *over the event times. In the case of proportional hazards, the overall HR is not affected by this weighting procedure. If, on the other hand, the HR changes over time, that is, the hazard rates are not proportional, then equal weighting may result in a non-representative HR, and may produce biased results [22]. It should be noted that the HR is averaged over the event times rather than over the follow-up time. It is unchanged if the time scale is changed without disturbing the ordering of events.

### Example

We applied some of the presented methods to breast cancer patients as time-varying effects have been reported, such as for nodal or hormone receptor status, [23-26]. We studied women with non-metastatic, operable breast cancer who underwent surgery between 1989 and 1993 at our institution, and who did not receive previous neoadjuvant treatment. Exclusion criteria included a previous history of breast carcinoma, concurrent contralateral breast cancer, and pathologic data missing. Follow-up was performed according to the European Good Clinical Practice requirements and consisted of regular physical examinations, and annual X-ray mammogram, and additional assessments in case of suspected metastases. Clinical and pathological characteristics were analyzed according to the hospital-recorded file at the time of treatment initiation. Pathological tumour size (≤ or > 20 mm) was measured on fresh surgical specimens. A modified version of the Scarff-Bloom-Richardson grading system was used (SBR grade I, II, or III). PVI (Yes, No) was defined as the presence of neoplastic emboli within unequivocal vascular lymphatic or capillary lumina in areas adjacent to the breast tumour. Exploratory immuno-histochemical analyses were performed on a tissue microarray (TMA) to assess hormone receptor (HRec) status (positive if ER-positive and/or progesterone receptor [PgR]-positive). ER and PgR expression levels were evaluated semi-quantitatively according to a standard protocol with cut-off values at 10% positive tumor cells. Her2 expression level was evaluated according to the Herceptest scoring system [27]. Mib1 expression level was evaluated semi-quantitatively. Information on all factors was available for 979 women (Table 1). The median follow-up time was 14 years (95% confidence interval: 13.7 - 14.2) and 264 women developed metastases.

**Table 1:** Characteristics of the study population.

	N	(%)
**Year of diagnosis**		
1989	231	23.6
1990	207	21.1
1991	182	18.6
1992	189	19.3
1993	170	17.4

**Metastases following surgery**		
Yes	264	27.0
No	715	73.0

**Age at diagnosis**		
≤ 40 years	76	7.8
> 40 years	903	92.2

**SBR Grade**		
Grade I	275	28.1
Grade II	444	45.3
Grade III	260	26.6

**Tumor size**		
≤ 20 mm	753	76.9
> 20 mm	226	23.1

**Lymph node involvement**		
No	554	56.6
Yes	425	43.4

**Peritumoral vascular invasion**		
No	700	71.5
Yes	279	28.5

**Hormone Receptor status**		
Both ER- and PR-	178	18.2
At least ER+ or PR+	801	81.8

**Her2 status**		
Positive	100	10.2
Negative	879	89.8

**Mib1 status**		
Negative	691	70.6
Positive	288	29.4

#### Working example

The prognostic factors were initially selected based on current knowledge regarding risk of metastases. They were next analyzed using a conventional Cox regression model; all were statistically significant at the 5% level in the univariate analyses, and were then entered onto a multivariate Cox model. The risk of metastases was increased for women with younger age compared to older age; grade II and III tumours compared to grade I tumours; large compared to small tumour sizes; lymph node involvement compared to no involvement; and PVI compared to no PVI (Additional file 1: Estimated log hazard ratios (log(HR)), and hazard ratios (HR = exp()) with 95% confidence intervals (95% CI) and p-values for model covariates when fitting a multivariate conventional Cox model and a Cox model with time-by-covariate interactions.). Based on this model, all variables, but hormone receptor, Her2 and Mib1 status, significantly affected the risk of metastases.

### Assessing non-proportionality: Graphical strategy

In the presence of a categorical variable, one can plot the Kaplan-Meier survival distribution, S(t), as a function of the survival time, for each level of the covariate. If the PH assumption is satisfied, the curves should steadily drift apart. One can also apply a transformation of the Kaplan-Meier survival curves and plot the function log(-log(S(t))) as a function of the log survival time, where log represents the natural logarithm function. If the hazards are proportional, the stratum specific log-minus-log plots should exhibit constant differences, that is be approximately parallel. These visual methods are simple to implement but have limitations. When the covariate has more than two levels, Kaplan-Meier plots are not useful for discerning non-proportionality because the graphs become to cluttered [10]. Similarly, although the PH assumption may not be violated, the log-minus-log curves are rarely perfectly parallel in practice, and tend to become sparse at longer time points, and thus less precise. It is not possible to quantify how close to parallel is close enough, and thus how proportional the hazards are. The decision to accept the PH hypothesis often depends on whether these curves cross each other. As a result, the decision to accept the PH hypothesis can be subjective and conservative [28], since one must have strong evidence (crossing lines) to conclude that the PH assumption is violated. In view of these limitations, some suggest providing standard errors to these plots [29]. This approach however can be computationally intensive and is not directly available in standard computer programs. Kaplan-Meier and log-minus-log plots are available from most standard statistical packages (Table 2).

**Table 2:** Statistical software

	*R/Splus*^©^	*SAS*^©^	*SPSS*^©^	*Stata*^©^
**Graphical checks**	survfit function	lifetest procedure	Survt command	sts command

**Time-by-covariate interactions**	programming required.	phreg procedure (definition of interactions)/test statement.	time program command (definition of interactions)/cox reg command.	tvc option/stcox command

**Scaled Schonfeld residuals**	cox.zph function	phreg procedure/ressch option	Not directly available/programming required	stphtest command

**Cumulative residuals**	Timereg/gof libraries/cum.residuals function	phreg procedure/assess statement/ph option	Not directly available/programming required	Not directly available/programming required

#### Working example (cont')

**Figure 1:**
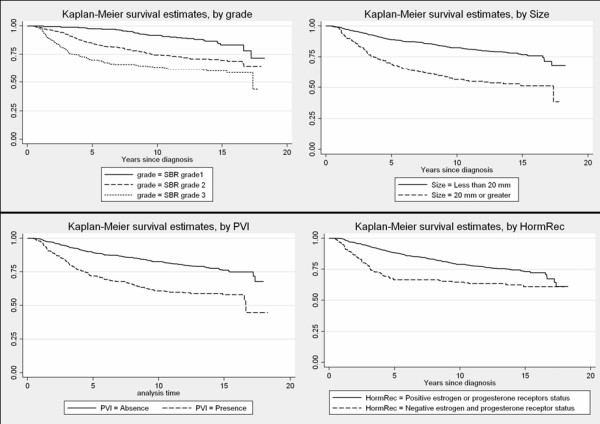
**Kaplan-Meier survival curves for SBR grade, tumour size, PVI, hormone receptor status**.

**Figure 2:**
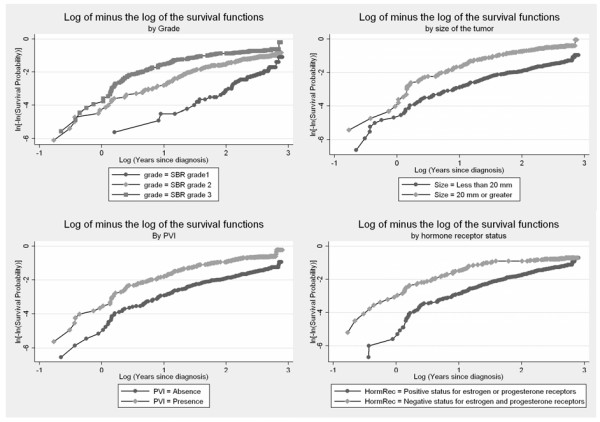
**Log(-log(survival)) curves as a function of time (log scale) for SBR grade, tumour size, PVI, hormone receptor status**.

Kaplan-Meier survival curves and log-minus-log plots are shown for some variables (Figures 1 and 2). The Kaplan-Meier survival curves appeared to steadily drift apart for all but the hormone receptor status, Her2 status, and mib1 status. The log-minus log plots looked approximately parallel for Age, size of the tumour, lymph node involvement, and PVI. Again, plots for the hormone receptor status, Her2 status, and mib1 status tended to indicate a violation of the PH assumption. There was also some suspicion with respect to the SBR grade.

### Assessing non-proportionality: Modelling and testing strategies

Graphical methods for checking the PH assumption do not provide a formal diagnostic test, and confirmatory approaches are required. Multiple options for testing and accounting for non-proportionality are available.

Cox proposed assessing departure from non-proportionality by introducing a constructed time-dependent variable, that is, adding an interaction term that involves time to the Cox model, and test for its significance [1]. Suppose one is interested in evaluating if some variable X has a time-varying effect. A time-dependent variable is created by forming an interaction (product) term between the predictor, X (continuous or categorical), and a function of time t (f(t) = t, t^2^, log(t), ...). Adding this interaction to the model (equation 2), the hazard then becomes:(5)

The hazard ratio is given by HR(t) = h_x+1_(t)/h_x_(t) = exp[β + γ.x.f(t)] for a unit increase in the variable X, and is time-dependent through the function f(t). If γ > 0 (γ < 0), then the HR increases (decreases) over time. Testing for non-proportionality of the hazards is equivalent to testing if γ is significantly different from zero. One can use different time functions such as polynomial or exponential decay but often very simple fixed functions of time such as linear or logarithmic functions are preferred [28]. This modeling approach also provides estimates of the hazard ratio at different time points since values t of time can be fitted into the hazard ratio function. Time-dependent variables provide a flexible method to evaluate departure from non-proportionality and an approach to building a model for the dependence of relative risk over time. This approach however should be used with caution. Indeed, if the function of time selected is mis-specified, the final model will not be appropriate. This is a disadvantage of this method over more flexible approach.

#### Working example (cont')

We created time-by-covariate interactions for each variable of the model, by introducing products between the variables and a linear function of time. As shown in Additional File 1 (Estimated log hazard ratios (log(HR)), and hazard ratios (HR = exp()) with 95% confidence intervals (95% CI) and p-values for model covariates when fitting a multivariate conventional Cox model and a Cox model with time-by-covariate interactions.), significant time-by-covariate interactions involved the SBR grade, hormone receptor status, Her2 status, and PVI (p < 0.05). Thus these results indicated that the hazard ratios associated with these factors were not constant over time. The parameters () associated with most interactions were negative, suggesting that the hazard ratios were decreasing over time. The estimated hazard ratio associated with an SBR grade II (versus grade I) as a function of time *t *was given by: HR(t) = exp(1.71 - 0.14t). Hazard ratios were 4.8, 3.6, and 2.7 at respectively 1, 3, and 5 years. Similarly, the estimated hazard ratio associated with the hormone receptor status was: HR(t) = exp(0.73 - 0.14t), that is hazard ratios of 1.8, 1.3, and 1.0 at respectively 1, 3, and 5 years. While the conventional Cox model did not show any significant effect for hormone receptors, Her2 and Mib1, these variables had a significant effect once time-by-covariate interactions were included.

Departure from non-proportionality can also be investigated using the residuals of the model. A residual measures the difference between the observed data, and the expected data under the assumption of the model. Schoenfeld residuals are calculated and reported at every failure time under the PH assumption, and as such are not defined for censored subjects [15,30]. They are defined as the covariate value for the individual that failed minus its expected value assuming the hypotheses of the model hold. There is a separate residual for each individual for each covariate. A smooth plot of the Schoenfeld residuals can then be used to directly visualize the log hazard ratio [15]. Assuming proportionality of the hazards, the Schoenfeld residuals are independent of time. Thus, a plot suggesting a non-random pattern against time is evidence of non-proportionality. Graphically, this method is more reliable and easier to interpret than plotting the log(-log(S(t)) function presented earlier. The presence of a linear relationship with time can be tested by performing a simple linear regression and a test trend. A slope significantly different from zero would be evidence against proportionality: an increasing (decreasing) trend would indicate an increasing (decreasing) hazard ratio over time. It is recommended to carefully look at the residual plot in addition to performing this test as some patterns may be apparent on the plots (quadratic, logarithmic), but remain undetected by the statistical test. Moreover, undue influence of outliers might become obvious [10]. Although, the method based on the smoothed Schoenfeld residuals provides time-dependent estimates, it can have some drawbacks [14,18]. The uncertainty estimates associated with the resulting time-dependent estimates can be difficult to use in practice, and the estimator provided may not have good statistical properties, such as consistency. Importantly, p-values resulting from trend tests based on the Schoenfeld residuals are obtained independently for each covariate of the model, assuming the Cox model is justified for the other covariates of the model; as such, results should be interpreted carefully. Tests based on the Schoenfeld residuals can be easily implemented in most standard statistical packages (Table 2).

#### Working example (cont')

**Figure 3:**
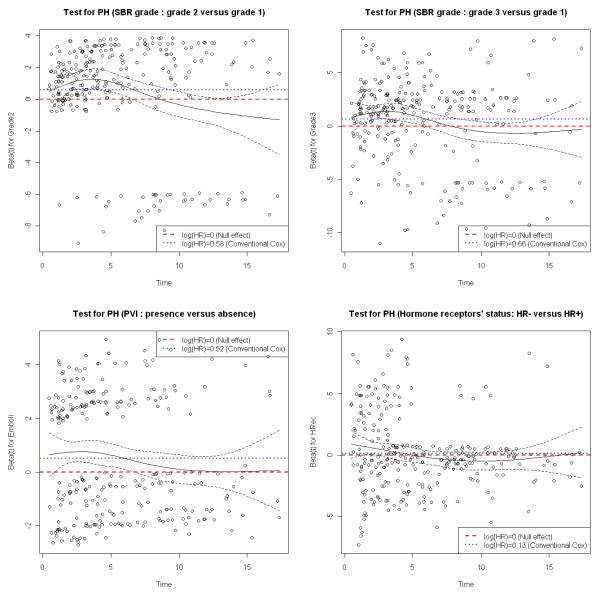
**Scaled Schoenfeld residuals for SBR grade, PVI, and hormone receptor status (with 95% confidence interval)**.

For each covariate, scaled Schoenfeld residuals were plotted over time, and tests for a zero slope were performed. The corresponding p-values, as well as the p-value associated with a global test of non-proportionality are reported in Table 3. The global test suggested strong evidence of non-proportionality (p < 0.01). Variables that deemed most likely to contribute to non-proportionality were the SBR grade (p < 0.01), PVI (p = 0.05) and hormone receptor status (p = 0.05). These numerical findings suggest a non constant hazard ratio for these variables. Residuals help visualizing the log hazard ratio  over time for each covariate (figure 3). We added dashed and dotted lines representing respectively the null effect (null log hazard ratio) and the averaged log hazard ratio estimated by the conventional Cox model. With respect to the SBR grade, the plots suggested strong effect over the first five years. This effect tended to diminish afterwards. Similarly, the impact of PVI changed over time, with again higher risks of metastases in the early years, and then this effect tended to vanish. Concerning hormone receptor status, plots suggested that a negative status increased the risk of metastases early on, and became protective afterwards.

**Table 3:** Test for non-proportionality based on the scaled Schoenfeld residuals from the conventional Cox model (see table 1).

Variable	p-value
Age	0.10
Grade II	<0.01
Grade III	<0.01
Size	0.32
Lymph node involvement	0.22
PVI	0.05
Hormone receptor	0.05
Her2	0.08
Mib1	0.07

**GLOBAL**	<0.01

The cumulative sum of Schoenfeld residuals, or equivalently the observed score process can also be used to assess proportional hazards [31]. Graphically, the observed score process is plotted versus time for each variable of the model, together with simulated processes assuming the underlying Cox model is true, that is, assuming proportional hazards. Any departure of the observed score process from the simulated ones is evidence against proportionality. These plots can then be used to assess when the lack of fit is present. In particular, an observed score well above the simulated process is an indication of an effect higher than the average one, and conversely. This method is particularly well illustrated in a recent publication by Cortese et al. [18]. Goodness-of-fit tests can be implemented based on the cumulative residuals. The cumulative residuals based approach overcomes some drawbacks encountered with the Schoenfeld residuals, since resulting estimators tend to have better statistical properties, and justified p-values are derived [14]. The cumulative residuals approach is implemented in some standard statistical packages (Table 2).

#### Working example (cont')

**Figure 4:**
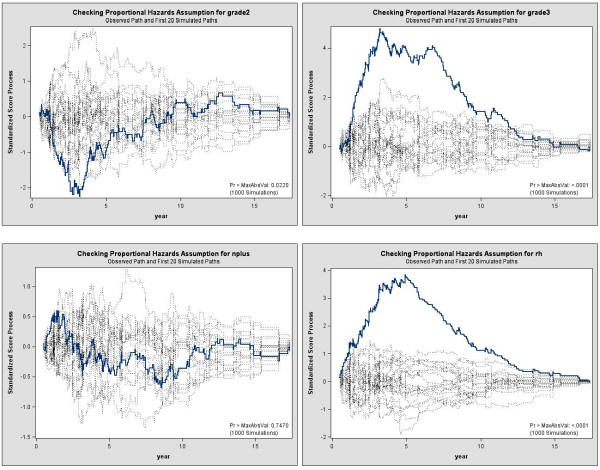
**Observed score process for SBR grade, lymph node involvement, and hormone receptor status (with 95% confidence interval)**.

Tests based on cumulative residuals are presented in Table 4. At the 5% significance level, test statistics suggest non-constant effect over time for the grade of the tumor, as well as the status of the hormone receptors, her2, and Mib1. For illustration, we also plotted the resulting score process for some variables (Figure 4). In accordance with the test statistics based on the cumulative residuals, we observe strong departure of the observed processes from the simulated curves under the model for the grade and hormone receptor status. These plots are particularly useful in identifying where the lack of fit is present. For example, the initial positive score process associated with hormone receptors, suggests that the effect of this variable is initially higher than the average effect, and thus lower than the average effect afterwards. That is, the risk of metastases is increased initially for women with both negative hormone receptors compared to the average risk, and decreased afterwards.

**Table 4:** Test for non-proportionality based on the Cumulative residuals from the conventional Cox model (see table 1).

Variable	p-value
Age	0.97
Grade II	0.02
Grade III	<0.01
Size	0.16
Lymph node involvement	0.75
PVI	0.11
Hormone receptor	<0.01
Her2	<0.01
Mib1	<0.01

Another simple approach for testing time-varying effects of covariates involves fitting different Cox models for different time periods. Indeed, although the PH assumption may not hold over the complete follow-up period, it may hold over a shorter time window. Unless there is an interest in a particular cut-off time value, two subsets of data can be created based on the median event time [10]. That is, a first analysis is conducted by censoring everyone still at risk beyond this time point, and a second one by considering only those subjects still at risk thereafter. In such case, the interpretation of the models is conditional on the length of the survival time, and results should thus be interpreted with caution. Even if the period of analysis is shortened, one should still ensure that the PH assumption is not violated within these reduced time periods. Moreover, since fewer event times are considered, analyses can suffer from a decreased power. Finally, although this method is particularly simple to implement and might provide sufficient information in some settings, that is if one is interested in a short time window, it should be noted that this method is not directly testing the PH assumption, and a different parametrization would be needed to perform such a test.

#### Working example (cont')

The median event time was 4.3 years. A Cox model was applied censoring everyone still at risk after 4.3 years, while only those subjects still at risk beyond this time point were included in another model (Additional file 2: Estimated hazard ratios (exp()) with 95% confidence intervals (95% CI) and p-values for model covariates in two independent Cox models for two different time periods.). All variables but age were statistically significant in the first model as negative hormone receptor status, positive Her2 status and Mib1 positive status were associated with an increased risk of metastases. In women still at risk past 4.3 years, younger age, greater tumor size, and lymph node involvement were associated with an increased risk of metastases. The effects of other variables have disappeared. Interestingly, hormone receptor negative status had a significant protective effect in this second model (HR = 0.5), while the first analysis suggested a significant increased risk for (HR = 1.7). Tests for non-proportionality based on the cumulative residuals suggested a persistent time-varying effect of the grade for the analysis restricted to the first 4.3 years.

It is also possible to account for non-proportionality by partitioning the time axis as proposed by Moreau et al. [32]. The time axis is partitioned and hazard ratios are then estimated within each interval. Thus, testing for non-proportionality is equivalent to testing if the time-specific HR are significantly different. Results can however sometimes be driven by the number of time intervals [33], and time intervals should thus be carefully selected.

Abandoning the assumption of proportional hazards, and as such, the Cox model, is another option. Indeed, other powerful statistical models are available to account for time-varying effects, including additive models, accelerated failure time models, regression splines models or fractional polynomials [33-36].

Finally, one can perform a statistical analysis stratified by the variable suspected to have a time-varying effect; this variable should be thus categorical or be categorized. Each stratum *k *has a distinct baseline hazard but common values for the coefficient vector β, that is, the hazard for an individual in stratum k is h_k_(t) = exp(βx) Stratifying assumes that the other covariates are acting in the same way in each stratum, that is, HRs are similar across strata. Although stratification is effective in removing the problem of non-proportionality and simple to implement, it has some disadvantages. Most importantly, stratification by a non-proportional variable precludes estimation of its strength and its test within the Cox model. Thus, this approach should be selected if one is not directly interested in quantifying the effect of the variable used for stratification. Moreover, a stratified Cox model can lead to a loss of power, because more of the data are used to estimate separate hazard functions; this impact will depend on the number of subjects and strata [10]. If there are several variables with time-varying risks, this would require the model to be stratified on these multiple factors, which again is likely to decrease the overall power.

## Discussion

While ensuring that the PH assumption holds is part of the modeling process, it is also useful in providing valuable information on time-varying effects. In our illustrative example, the conventional Cox model suggested that all factors but HRec, Her2, and Mib1 status were strong prognostic factors of metastases. Additional tests indicated that the PH assumption was not satisfied for some variables of the model. Tumour grade had a significant time-varying effect, but although its effect diminished over time, it remained strong. According to the conventional model hormone receptor status did not significantly impact relapses. Additional tests provided strong evidence of a time-varying effect. Importantly, both tests based on residuals suggested that negative hormone receptor status increased the risk of metastases early but became protective thereafter, in accordance with the analysis partitioned on event time. This reversal of effect may explain the non-significant averaged hazard ratio provided by the conventional Cox model and reported earlier [26].

Applying a Cox model without ensuring that its underlying assumptions are validated can lead to negative consequences on the resulting estimates [28,37]. For variables not satisfying the non-proportionality assumption, the power of the corresponding tests is reduced, that is, we are less likely to conclude for a significant effect when there is actually one. If the hazard ratio is increasing over time, the estimated coefficient assuming PH is overestimating at first and underestimating later on. For those variables of the model with a constant hazard ratio, the power of tests is also reduced as a consequence of an inferior fit of the model.

Once non-proportionality is established, time-dependency can be accounted for in different ways. The strategy will depend on the study objectives. If there is no interest in longer time periods, one can shorten the follow-up time as non-proportionality is less likely to be an issue on short time intervals. If there is no particular interest in the variable with the time-varying effect, one could stratify on this variable in the statistical analysis, however no association between the stratification variable and survival can be tested. If one wants to describe the effect of the variable over time, it is possible to rely on time by covariate interactions or on plots of residuals to estimate of relative risks at different time points. Methods to test and account for non-proportionality are available in most standard statistical software (Table 2).

It is difficult to propose definite guidelines for the best strategy for testing for non-proportionality. Each method has its advantages and limitations, and depending on the study objective some approaches might be preferred. Before performing statistical modeling, the study objectives should be clearly stated in advance, as well as the statistical tests that will be employed. Departure from non-proportionality can be investigated using graphical and numerical approaches. Plotting methods involve visualizing the Kaplan-Meier survival curves for the variable tested for non-proportionality. This graphical method requires categorical variables, and is particularly appropriate for binary data; however they do not provide formal diagnostic tests. Numerical tests involve for example testing for covariate-by-time interactions or for the presence of a trend in the residuals of the model. Including a covariate-by-time interaction is particularly simple within the Cox model; however, results are strongly dependent on the choice of the functional form of the time function. Tests based on cumulative residuals tend to have better statistical properties than those based on the Schoenfeld residuals. As a result, performing a test based on the cumulative residuals seems to be a more powerful approach in detecting covariates with time-varying effects.

Note that the Cox model involves multiple types of residuals including the martingale, deviance, score and Schoenfeld residuals, which can be particularly useful as additional regression diagnostics for the Cox model. Martingale residuals are useful for determining the functional form of a covariate to be included in the model and deviance residuals can be used to examine model accuracy. Additional details can be found in [10,11].

Statistical testing raises the issue of power, that is, the ability of tests to find true effects. We have seen for example that some simple strategies, such as shortening the observation period can suffer from reduced power as fewer events are considered. This might be a limitation with small datasets. Simulations have shown that stratified Cox modeling usually leads to wider confidence intervals, that is, reduced power compared to unstratified analysis [38]. Statistical tests for time-varying effects have different power to detect non-proportionality. It has been shown that tests requiring partitioning of the failure time have less power than other tests, while tests based on time-dependent covariates or on the Schoenfeld residuals have equally good power to detect non-proportionality in a variety of non-proportional hazards and are practically equivalent [17]. The issue of power naturally leads to the question of sample size. Clinical trials are usually designed with just enough power to detect the treatment effect. In this context, one should not expect to have enough details about the actual shape of the HR over time. Assuming a trial designed with an 80% power to detect a treatment effect, Therneau and Grambsch showed that the test based on the residuals was able to detect non-proportionality, but could not distinguish between a linear and a discrete increase of the hazard ratio over time [10]. Observational studies are usually designed for exploratory analyses and do not rely on a formal estimation of the sample size. There might not always be enough power to detect a specific time trend. The question of lack of power should not be interpreted as an argument against testing for non-proportionality. Just as any other statistical model, one should ensure that major assumptions are not violated.

Since its original publication in 1972, the Cox proportional-hazards model has gained widespread use and has become a popular tool for the analysis of survival data in medicine. After performing an online search, we found that the original paper by Cox had been cited approximately 25, 000 times, with about 8, 000 citations in oncology papers [4]. While time dependency has been accounted for and reported in oncology publications, such as in breast or colon cancer studies [26,33,39-42,42], the verification of the PH assumption is unfortunately far from being systematic. In a 1995 review of five clinical oncology journals including about 130 papers, Altman et al. reported that only 2 out of the 43 papers which relied on a Cox model, mentioned that the PH assumption was verified [2]. Similarly, about ten years later Mathoulin et al. assessed the quality of reporting of survival events in randomized clinical trials in eight general or cancer medical journals [3]. The authors reported that only one of the 64 papers that used a Cox model mentioned verifying the PH assumption.

Our objective was to familiarize the reader with the PH assumption. We also highlighted that detecting and accounting for time-varying effects provide insights on some specific time patterns and valuable biological information that could be missed otherwise. Given the possible consequences on parameter estimates, checking the proportionality of hazards should be an integral part of a survival analysis based on a Cox model. In the presence of variables with time-varying risks, plots should be used to augment the results and indicate where non-proportionality is present. This seems particularly appropriate in the context of oncology studies, as long follow-ups are common and non-constant hazards have already been reported.

## Conclusions

Investigating time-varying effects should be an integral part of Cox survival analyses. Detecting and accounting for time-varying effects provide insights on some specific time patterns, and on valuable biological information that could be missed otherwise.

## Competing interests

The authors declare that they have no competing interests.

## Authors' contributions

CB conceived the study, performed the statistical analysis and drafted the manuscript. GMG carried out the immunoassays. MD provided clinical expertise in oncology. CTL provided clinical expertise in surgery. VB was responsible of the datamanagement. SMP participated in the design of study. All authors read and approved the final manuscript.

## Pre-publication history

The pre-publication history for this paper can be accessed here:

http://www.biomedcentral.com/1471-2288/10/20/prepub

## Supplementary Material

Additional File 1Estimated log hazard ratios (log(HR)), and hazard ratios (HR = exp()) with 95% confidence intervals (95% CI) and p-values for model covariates when fitting a multivariate conventional Cox model and a Cox model with time-by-covariate interactions.Click here for file

Additional File 2Estimated hazard ratios (exp()) with 95% confidence intervals (95% CI) and p-values for model covariates in two independent Cox models for two different time periods.Click here for file
