# Supplementary material for: Variables with time-varying effects and the Cox model: Some statistical concepts illustrated with a prognostic factor study in breast cancer
Source: BMC Med Res Methodol. 2010 Mar 16;10:20. doi: 10.1186/1471-2288-10-20 (PMC2846954; doi:10.1186/1471-2288-10-20)
Supplement: Additional File 1 — Estimated log hazard ratios (log(HR)), and hazard ratios (HR = exp()) with 95% confidence intervals (95% CI) and p-values for model covariates when fitting a multivariate conventional Cox model and a Cox model with time-by-covariate interactions. [file 1471-2288-10-20-S1.DOC]

**Additional file 1**. Estimated log hazard ratios (log(HR)), and hazard ratios (HR = exp($\hat{\beta}$)) with 95% confidence intervals (95% CI) and p-values for model covariates when fitting a multivariate conventional Cox model and a Cox model with time-by-covariate interactions.

|  | **Conventional Cox model** | | | | Cox model with time-by-covariate interactions | | | |
| --- | --- | --- | --- | --- | --- | --- | --- | --- |
| Variable (Reference) | **log(HR)** | **HR = exp($\hat{\beta}$)** | **95% CI** | **p-value** | log(HR) | HR = exp($\hat{\beta}$) | 95% CI | p-value |
| Age (ref: older than 40) | 0.59 | 1.80 | (1.25; 2.57) | <0.01 | 0.18 | 1.19 | (0.65; 2.18) | 0.56 |
| Grade II (ref: Grade I)Grade III (ref: Grade I) | 0.58  0.66 | 1.78  1.94 | (1.22; 2.61)  (1.24; 3.04) | <0.01 <0.01 | 1.711.92 | 5.546.82 | (2.34; 13.12)(2.61; 17.82) | <0.01<0.01 |
| Size (ref: ≤ 20mm) | 0.67 | 1.96 | (1.51; 2.54) | <0.01 | 0.85 | 2.34 | (1.50; 3.66) | <0.01 |
| Lymph node involvement (ref: None) | 0.67 | 1.96 | (1.49; 2.57) | <0.01 | 0.44 | 1.56 | (0.97; 2.48) | 0.06 |
| PVI (ref: none) | 0.52 | 1.68 | (1.30; 2.18) | <0.01 | 0.91 | 2.48 | (1.58; 3.88) | <0.01 |
| Hormone receptor (ref: ER+ or PR+) | 0.13 | 1.14 | (0.80; 1.63) | 0.47 | 0.73 | 2.07 | (1.11; 3.84) | 0.02 |
| Her2 status (ref: negative) | 0.31 | 1.37 | (0.93; 2.01) | 0.11 | 0.96 | 2.60 | (1.35; 5.03) | <0.01 |
| Mib1 status (ref: negative) | 0.17 | 1.18 | (0.89; 1.57) | 0.25 | 0.59 | 1.80 | (1.10; 2.95) | 0.02 |
| Age * time | - | - | - | - | 0.07 | 1.08 | (0.98; 1.18) | 0.10 |
| Grade II * time | - | - | - | - | -0.14 | 0.87 | (0.79; 0.96) | <0.01 |
| Grage III * time | - | - | - | - | -0.16 | 0.85 | (0.75; 0.96) | <0.01 |
| Size * time | - | - | - | - | -0.03 | 0.97 | (0.90; 1.04) | 0.36 |
| Lymph node involv.* time | - | - | - | - | 0.03 | 1.03 | (0.97; 1.10) | 0.36 |
| PVI * time | - | - | - | - | -0.07 | 0.93 | (0.87; 0.99) | 0.03 |
| Hormone Receptor status * time | - | - | - | - | -0.14 | 0.87 | (0.77; 0.99) | 0.03 |
| Her2 status * time | - | - | - | - | -0.17 | 0.85 | (0.73; 0.99) | 0.04 |
| Mib1 status * time | - | - | - | - | -0.08 | 0.93 | (0.85; 1.00) | 0.07 |
