# Supplementary material for: Variables with time-varying effects and the Cox model: Some statistical concepts illustrated with a prognostic factor study in breast cancer
Source: BMC Med Res Methodol. 2010 Mar 16;10:20. doi: 10.1186/1471-2288-10-20 (PMC2846954; doi:10.1186/1471-2288-10-20)
Supplement: Additional File 2 — Estimated hazard ratios (exp()) with 95% confidence intervals (95% CI) and p-values for model covariates in two independent Cox models for two different time periods. [file 1471-2288-10-20-S2.DOC]

**Additional file 2**. Estimated hazard ratios (exp($\hat{\beta}$)) with 95% confidence intervals (95% CI) and p-values for model covariates in two independent Cox models for two different time periods.

|  | **Analysis 1:**  **Event time shorter than 4.3 years (*)** | | | **Analysis 2:** Event time longer 4.3 years (**) | | |
| --- | --- | --- | --- | --- | --- | --- |
| Variable (Reference) | HR | 95% CI | **p-value** | HR | 95% CI | p-value |
| Age (ref: older than 40) | 1.41 | (0.86; 2.31) | 0.17 | 2.33 | (1.38; 3.92) | <0.01 |
| Grade II (ref: Grade I)Grade III (ref: Grade I) | 6.13  7.01 | (2.20; 17.1)  (2.41; 20.4) | <0.01  <0.01 | 1.301.36 | (0.84; 2.00)  (0.76; 2.43) | 0.240.31 |
| Size (ref: ≤ 20mm) | 2.00 | (1.40; 2.86) | <0.01 | 1.90 | (1.30; 2.79) | <0.01 |
| Lymph node involvement (ref: None) | 1.71 | (1.16; 2.53) | <0.01 | 2.07 | (1.42; 3.02) | <0.01 |
| PVI (ref: none) | 2.02 | (1.40; 2.91) | <0.01 | 1.38 | (0.94; 2.00) | 0.10 |
| Hormone receptor (ref: ER+ or PR+) | 1.71 | (1.10; 2.69) | 0.02 | 0.51 | (0.25; 1.02) | 0.06 |
| Her2 status (ref: negative) | 1.75 | (1.12; 2.74) | 0.01 | 0.76 | (0.33; 1.73) | 0.51 |
| Mib1 status (ref: negative) | 1.47 | (1.01; 2.15) | 0.05 | 0.96 | (0.62; 1.50) | 0.86 |

(*) In this analysis, data on women still at risk after 4.3 years were censored. The cut-off of 4.3 years corresponds to the median event time and allows maximizing the number of events in each analysis.

(**) In this analysis, only women at risk past 4.3 years were considered.
